# Diagnostic tests to predict outcome in patients with implantable cardioverter defibrillators: it is not that simple!

**DOI:** 10.1007/s12471-014-0597-x

**Published:** 2014-09-04

**Authors:** A. H. Maass

**Affiliations:** Department of Cardiology, Thoraxcenter, University of Groningen, University Medical Center Groningen, PO Box 30.001, 9700 RB Groningen, the Netherlands

Implantable cardioverter defibrillators (ICDs) are effective for both primary and secondary prevention of sudden cardiac death. For patients with reduced left ventricular ejection fraction (LVEF) caused by ischaemic or non-ischaemic aetiology, ICDs have received a class I indication in the recent European and American guidelines (reviewed by Maass and Van Veldhuisen [1]). Selection of patients has become simple, as we have to rely mostly on LVEF with few extra criteria such as functional class. The disadvantage of simple criteria is the offset of high sensitivity with low specificity. Using the current guidelines, many patients who receive ICDs especially for primary prevention will never receive appropriate ICD therapies. Utilisation of ICDs according to the guidelines might be offset by severe complications, not only peri-operatively but also in the long term. Inappropriate ICD therapy, lead problems, and primary or secondary infection of ICD systems can all lead to morbidity and mortality. Furthermore, cost-effectiveness of primary prevention ICDs has recently been questioned. The DO-IT registry will investigate outcomes of primary prevention ICDs in the Netherlands and we are eagerly awaiting these results to justify implantations in this situation. Current data suggest that real-life ICD therapy in Dutch centres is beneficial even though certain patients might benefit less [2].

Which diagnostic tests can be used to govern ICD implantations? Risk prediction is even more important for non-ischaemic cardiomyopathy as the evidence for prophylactic ICD implantation is rather weak. Tests should be easy to perform and results should be unambiguous.

Currently, LVEF is the gold standard to indicate a patient for ICD implantation. The test that should be used to quantify LVEF, however, is not mentioned in the guidelines and results from echocardiography, nuclear imaging or magnetic resonance imaging might be divergent.

Simple clinical characteristics such as functional class and history of syncope are easy to record but not unambiguous. Functional class is very subjective and neurocardiogenic syncope is very common and occurs often without underlying cardiac pathology. In addition to functional class, renal insufficiency, chronic obstructive pulmonary disease, age and sex all influence risk of sudden death. The predictive value, however, is offset by an increase in all-cause mortality, and risk reduction by ICDs is low in high-risk populations. High-risk patients derive no benefit from non-resynchronisation ICD therapy [3]. Even though increasing age leads to increase in all-cause mortality, age per se is a poor predictor of outcome of ICD therapy [4].

Registration of spontaneous non-sustained ventricular tachycardias (VTs) via Holter monitoring is also simple but sensitivity might also be low with a single 24-hour recording. Furthermore, the prognostic value of spontaneous VTs has only been demonstrated in depressed ejection due to ischaemic aetiology whereas results in patients with non-ischaemic cardiomyopathy are less clear. Inducibility of monomorphic VTs by programmed electrical stimulation might be of predictive value but results from studies are not consistent especially for non-ischaemic cardiomyopathy and this test is invasive and not particularly easy to perform outside of specialised centres.

Genetics are currently used to confirm the diagnosis in familial heart diseases such as hypertrophic and dilated cardiomyopathy. In dilated cardiomyopathy, common mutations in Dutch patients have malignant phenotypes such as lamin A/C and phospholamban. In these patients, ICD implantation can be useful even if decline in LVEF has not yet reached 35%. Most likely, the near future will reveal common disease-modifying polymorphisms that render their carriers at increased risk of sudden death if they develop heart disease. We will have to wait for identification of such genetic modifiers but once they are known testing would fulfil the criterion of simplicity and possibly unambiguity.

Several parameters of the electrocardiogram have been used to predict risk: resting heart rate, heart rate recovery, heart rate variability or turbulence, QT dispersion or microvolt T wave alternans (MTWA). In this issue of the journal, Kraaier et al. test whether exercise MTWA can be used to identify patients at high risk of death or appropriate ICD shocks [5]. MWTA testing did not predict mortality but test ineligibility was a significant predictor of mortality. A significant portion of patients were ineligible for this test. Atrial fibrillation is an exclusion criterion and is common in heart failure patients indicated for ICD. Furthermore, MWTA testing involves an exercise protocol. This test, therefore, does not qualify as simple. The current study also shows that MWTA is also not unambiguous.

Where are we headed with prophylactic ICD implantations? Cardiology has recently seen a dramatic increase in the use of scores to govern diagnostics and treatment. Oral anticoagulation therapy is governed by the CHADS-VASc score, cardiothoracic surgery risk is evaluated with the EUROSCORE, and we use the GRACE score to evaluate risk in patients with acute coronary syndromes. Why are we not using a risk score to predict efficacy of ICDs? We urgently need more than LVEF to decide on which patients should receive an ICD. In the current situation, we might implant patients at low risk who might never receive adequate ICD therapies and are at risk to develop serious complications. On the other hand, patients with an LVEF presumed to be above 35% might be at risk of sudden cardiac death and not receive this life-saving therapy. The prognosis of patients with heart failure with preserved ejection fraction (HFPEF) is not much better than that of patients with depressed ejection fraction [1]. Sudden cardiac death is also common in HFPEF patients. We really need additional diagnostic tests and risk scores to govern ICD implantation irrespective of ejection fraction or at least with it being just one of the factors taken into consideration.
